# Local Differences in Network Organization in the Auditory and Parietal Cortex, Revealed with Single Neuron Activation

**DOI:** 10.1523/JNEUROSCI.1385-24.2025

**Published:** 2025-01-31

**Authors:** Christine F. Khoury, Michael Ferrone, Caroline A. Runyan

**Affiliations:** ^1^Department of Neuroscience, University of Pittsburgh, Pittsburgh, Pennsylvania 15260; ^2^Center for the Neural Basis of Cognition, University of Pittsburgh, Pittsburgh, Pennsylvania 15260

**Keywords:** auditory cortex, cortical circuits, inhibitory neurons, posterior parietal cortex, single neuron optogenetics

## Abstract

The structure of local circuits is highly conserved across the cortex, yet the spatial and temporal properties of population activity differ fundamentally in sensory-level and association-level areas. In the sensory cortex, population activity has a shorter timescale and decays sharply over distance, supporting a population code for the fine-scale features of sensory stimuli. In the association cortex, population activity has a longer timescale and spreads over wider distances, a code that is suited to holding information in memory and driving behavior. We tested whether these differences in activity dynamics could be explained by differences in network structure. We targeted photostimulations to single excitatory neurons of layer 2/3, while monitoring surrounding population activity using two-photon calcium imaging. Experiments were performed in the auditory (AC) and posterior parietal cortex (PPC) within the same mice of both sexes, which also expressed a red fluorophore in somatostatin-expressing interneurons (SOM). In both cortical regions, photostimulations resulted in a spatially restricted zone of positive influence on neurons closely neighboring the targeted neuron and a more spatially diffuse zone of negative influence affecting more distant neurons (akin to a network-level “suppressive surround”). However, the relative spatial extents of positive and negative influence were different in AC and PPC. In PPC, the central zone of positive influence was wider, but the negative suppressive surround was more narrow than in AC, which could account for the larger-scale network dynamics in PPC. The more narrow central positive influence zone and wider suppressive surround in AC could serve to sharpen sensory representations.

## Significance Statement

The structure of local circuits is conserved across the cortex, and yet local processing goals vary across the sensorimotor hierarchy, from sensory perception to the control of behavior. It has been unclear whether these differences in function require different network organization. To probe the spatial structure of networks in sensory and association-level cortex, we photostimulated single excitatory neurons and measured the effects on local population activity in mice. Stimulations triggered a centered, positive activity change in neighboring neurons and a surrounding, suppressive change in more distant neurons. The relative sizes of the center and surround differed across areas, suggesting that network structure is tailored for sharper, more restricted activity in the sensory cortex and more dense network activity in the association cortex.

## Introduction

Cortical circuits consist of a rich diversity of cell types, which interact to transform incoming signals by shaping receptive field properties (Adesnik et al., 2012; Lee et al., 2012; Wilson et al., 2012), controlling response gain (Atallah et al., 2012; Wilson et al., 2012; Veit et al., 2022), and amplifying (Ko et al., 2011; Cossell et al., 2015; Akitake et al., 2023; Oldenburg et al., 2024) or sparsening responses to stimuli (Chettih and Harvey, 2019; Oldenburg et al., 2024). Circuit dissection experiments in the sensory cortex have begun to characterize these local network interactions and their corresponding computational impacts (Yoshimura and Callaway, 2005; Yoshimura et al., 2005; Fino and Yuste, 2011; Packer and Yuste, 2011; Chettih and Harvey, 2019; Akitake et al., 2023; Oldenburg et al., 2024). Excitatory neurons form functionally specific recurrent subnetworks in the sensory cortex, where neurons with similar response properties tend to form stronger, bidirectional synaptic connections (Yoshimura et al., 2005; Ko et al., 2011; Perin et al., 2011; Li et al., 2012; Cossell et al., 2015). In vivo, two-photon optogenetic stimulation of single neurons combined with simultaneous calcium imaging of the local population can be used to characterize “influence maps” of single neurons’ activity on the surrounding population. These influence maps are the net result of triggered activation across excitatory and inhibitory connections and their synaptic weights across the network, possibly even including longer-range interactions with unobserved neurons in different layers or brain regions. Although this approach generally cannot reveal the actual synaptic pathways from the stimulated neuron to the neurons it affects, the influence map represents the functional, network-level impact of a single neuron on the activity in the local population (Kwan and Dan, 2012; Chettih and Harvey, 2019; Oldenburg et al., 2024). This in vivo influence mapping approach, applied in primary sensory cortex, suggests that recurrent excitation can serve as signal amplification or to sparsen stimulus-evoked activity, depending on the details of experimental conditions, such as the number of neurons being stimulated, their tuning preferences, and the sensory context (Chettih and Harvey, 2019; Akitake et al., 2023; Oldenburg et al., 2024). The functional impact of recurrent excitatory interactions is thus state dependent in sensory cortex.

Inhibitory interneurons are key in modulating both local network states and the impacts of feedforward and recurrent excitation. Through cell-type–specific interactions (Yoshimura and Callaway, 2005; Lee et al., 2012; Wilson et al., 2012; Pfeffer et al., 2013; Pi et al., 2013; Green et al., 2023), inhibitory interneurons can profoundly shift population activity dynamics and also shape the response properties of individual neurons. For example, somatostatin-expressing (SOM) interneurons directly inhibit both other inhibitory interneurons and excitatory pyramidal cells (Pfeffer et al., 2013; Condylis et al., 2022), refining receptive fields (Adesnik et al., 2012; Wilson et al., 2012; Kato et al., 2017), decorrelating population activity at slow timescales (Chen et al., 2015), and modulating high-frequency oscillations (Veit et al., 2017). SOM neurons tend to be highly coordinated as populations (Karnani et al., 2016; Knoblich et al., 2019; Khoury et al., 2022), and so they are particularly well suited to modulate local network states in the cortex.

Previously, cellular and synaptic-level circuit manipulations have characterized the organization of local circuits and the local influence of individual neurons in the cortex almost exclusively in sensory regions. So it is not yet known how the structural and functional organization of local circuits may differ across cortical regions with different computational goals. In sensory cortex, topographically organized inputs from core sensory nuclei of the thalamus generate or maintain tuning for specific stimulus features, such as retinotopic location or the orientation of an edge in the primary visual cortex (Reid and Alonso, 1995) or best frequency across the tonotopic axis in the primary auditory cortex. These tuning preferences are then further shaped by local excitatory and inhibitory interactions (Somers et al., 1998; Troyer et al., 1998). In association-level areas such as the posterior parietal cortex (PPC), sensory inputs across several modalities are flexibly combined (Olcese et al., 2013; Song et al., 2017; Mohan et al., 2018) toward sensorimotor decisions (Shadlen and Newsome, 1996; Harvey et al., 2012; Tseng et al., 2022; Zhou et al., 2023). The computations that integrate sensory information across modalities and across time in areas like PPC may depend on more spatially distributed networks compared with those needed to encode fine-scale sensory features in topographically organized sensory areas (Markowitz et al., 2015; Safavi et al., 2018). Systematic changes in neuronal morphology (Elston, 2000) and in the timescale of activity in individual neurons (Murray et al., 2014) and in populations (Honey et al., 2012; Runyan et al., 2017; Safavi et al., 2018; Khoury et al., 2022) across the cortical hierarchy have led to hypotheses that the contribution of recurrent connections to neuronal activity becomes systematically stronger across the cortical hierarchy, in support of cognitive processes requiring temporal integration (Chaudhuri et al., 2015; Hasson et al., 2015).

We hypothesized that the density and spatial scale of recurrent connections could explain differing spatial and temporal scales of activity reported across the cortex and across cell types (Khoury et al., 2022). If this is the case, the activation of single excitatory neurons should influence the local population more widely in the association cortex than in the sensory cortex. To test this, we targeted photostimulations to individual excitatory neurons in the auditory cortex (AC) and posterior parietal cortex (PPC) while monitoring spike-related activity in neighboring excitatory (E) and SOM neurons. In both regions, the stimulations positively influenced a narrow, central zone of neighboring neurons, and negatively influenced a diffuse, surrounding zone including more distant neurons. The relative sizes of these two zones differed across areas, reflecting the observed population activity differences in AC and PPC. In PPC, the central positive zone of influence was wider, and the surrounding negative zone was more narrow than in AC. As a consequence, excitation can spread more easily across the network in PPC, while in AC, the spread of excitation is restricted by the stronger suppressive surround.

## Materials and Methods

### Experimental model and subject details

All procedures were approved by the University of Pittsburgh Institutional Animal Care and Use Committee. Homozygous SOM-Cre mice (Sst-IRES-Cre, JAX Stock #013044) were crossed with homozygous Ai14 mice (RCL-tdT-D, JAX Stock #007914) obtained from the Jackson Laboratory, and all experiments were performed in the F1 generation, which expressed tdTomato in SOM+ neurons. Mice were group housed in cages with between two and four mice. Adult (8–24 weeks) male and female mice were used for experiments (4 male, 6 female). Mice were housed on a reversed 12 h light/dark cycle, and all experiments were performed in the dark (active) phase.

### Surgery

Mice were anesthetized with isoflurane (4% for induction, and 1–2% maintenance during surgery) and mounted on a stereotaxic frame (David Kopf Instruments). Ophthalmic ointment was applied to cover the eyes (Henry Schein). Dexamethasone was injected 12–24 h prior to surgery, and carprofen and dexamethasone (Covetrus) were injected subcutaneously immediately prior to surgery for pain management and to reduce the inflammatory response. Two 2 × 2 mm craniotomies were made, over left AC (centered on the temporal ridge, with the posterior edge along the lambdoid suture) and PPC (centered at 2 mm posterior and 1.75 mm lateral to the bregma). One to four evenly spaced ∼60 nl injections of a viral mixture were made in each cranial window, centered on the same coordinates listed above. The viral mixture contained AAV1-synapsin-l-GCamp6f (Addgene, MA stock #100837; Chen et al., 2013) and AAV9-CAMKII-mScarlet-C1V1-KV2.1 (Addgene, MA stock # 124650), mixed 1:1 and diluted to a titer of ∼1 × 10^12^ vg/ml using sterile PBS, and a micromanipulator (QUAD, Sutter) was used to target injections ∼250 µm under the dura at each site, where ∼60 nl virus was pressure-injected over 5–10 min. pAAV-CamKIIa-C1V1(t/t)-mScarlet-KV2.1 was a gift from Christopher Harvey (Addgene viral prep # 124650-AAV9; http://n2t.net/addgene:124650; RRID:Addgene_124650; Chettih and Harvey, 2019). Pipettes were not removed until 5 min postinjection to prevent backflow. Dental cement (Parkell) sealed a glass coverslip (3 mm) over a drop of Kwik-Sil (World Precision Instruments) on the craniotomy. Using dental cement, a one-sided titanium headplate was attached to the right hemisphere of the skull. After mice had recovered from the anesthesia, they were returned to their home cages and received oral carprofen tablets (Fisher Scientific) daily for 3 d postsurgery.

### Two-photon microscope

Imaging and photostimulation were performed entirely within layer 2/3, using a resonant scanning two-photon microscope (Ultima 2Pplus, Bruker). Images were collected at a 30 Hz frame rate and 512 × 512 pixel resolution through a 16× water immersion lens (Nikon CF175, 16X/0.8 NA). On separate days in the same mice, either AC or PPC was imaged at a depth between 150 and 300 µm, corresponding to layers 2/3 of the cortex. For AC imaging, the objective was rotated 35–45° from vertical, and for PPC imaging, it was rotated to 5–15° from vertical, matching the angle of the cranial window implant. AC and PPC imaging was performed on alternating days in the same mice.

Excitation light was provided by a femtosecond infrared laser (InSight X3, Spectra-Physics) tuned to 920 nm. Photostimulation was achieved by controlling a 1,045 nm beam (secondary output from the InSight X3) with an independent set of galvanometers. The 920 and 1,045 nm beams were combined with a dichroic (Chroma, ZT1040crb). Green and red wavelength emission light was separated through a 565 nm low-pass filter before passing through bandpass filters (Chroma, ET525/70 and ET595/50). PrairieView software (v5.5, Bruker) was used to control the microscope.

### Photostimulation

Excitatory neurons expressing mScarlet could be distinguished from the tdTomato + SOM neurons by the subcellular localization of the fluorophores as well as by collecting images at 800 nm excitation, at which mScarlet is more strongly excited than tdTomato (Drobizhev et al., 2009). A total of 16–47 mScarlet+ neurons were selected as targets in each experiment. The number depended on expression levels, as cells were chosen to have robust mScarlet and GCaMP expression levels. Then, 5–10 “control” targets not expressing mScarlet were also chosen, so that the specificity of stimulation effects could be assessed. The secondary pair of galvanometers was used to direct spiral scans of the 1,045 nm beam over individual neurons.

Spirals (13 µm in diameter) were directed to each target at 250 Hz for 100 ms, at 20–50 mW. These parameters were chosen to elicit reliable responses in the targeted neurons, while limiting stimulation power (Chettih and Harvey, 2019; Russell et al., 2022; Oldenburg et al., 2024). At the beginning of each experiment, one target was chosen for stimulation at a range of excitation powers. The lowest power that could reliably activate the cell was selected for the rest of the experimental session. For influence mapping, the full set of individual mScarlet+ and control targets were stimulated in pseudorandom order with a 1 s interstimulus interval. The 1 s interstimulus interval was chosen to allow for a large number of trial repeats within the imaging session, while also allowing for enough time for the neural responses elicited by each stimulation to return to baseline before the next stimulation. At least 100 trial repeats were performed, as this is the number of stimulations that could be accomplished within a typical 60–90 min imaging session. Different pseudorandom orderings were used across trial repeats, to avoid driving plasticity-related changes in network structure.

### Behavioral monitoring

Throughout the imaging experiments, mice ran voluntarily on a spherical treadmill. Running velocity was monitored on pitch, roll, and yaw axes using two optical sensors (ADNS-98000, Tindie) held adjacent to the spherical treadmill. A microcontroller (Teensy, 3.1, Adafruit) communicated with the sensors, demixing their inputs to produce one output channel per rotational axis using custom code. Outputs controlling the galvanometers were synchronized with running velocity using a digital oscilloscope (WaveSurfer).

### Sound stimuli

In auditory cortex imaging sessions, either immediately before or following influence mapping, the same field of view was imaged while presenting sinusoid amplitude-modulated (10 Hz) pure tone stimuli. One magnetic speaker was pointed to the ear contralateral to the imaging hemisphere (MF1-S, Tucker-Davis). Tones were played at frequencies of 4, 8, 12, 16, 24, and 32 kHz for 1 s each. Sounds were played at a single intensity (70 dB). Sound responsiveness of each neuron was calculated based on the mean *z*-scored deconvolved activity of each neuron aligned on sound onset. We chose not to measure sound frequency tuning in the posterior parietal cortex (PPC) because of its unreliable sound-evoked activity in passive listening contexts.

### Image processing

For each field of view, the raw calcium movies were concatenated for motion correction, cell body identification, and fluorescence and neuropil extraction. These processing steps were performed using Suite2p 0.9.3 in Python (Pachitariu et al., 2017). Suite2p first registered images to account for brain motion, and clustered neighboring pixels with similar time courses into regions of interest (ROIs). ROIs were manually curated using the Suite2p GUI, to ensure that only cell bodies as opposed to dendritic processes were included in analysis, based on morphology. Cells expressing tdTomato (SOM cells) were identified using a threshold applied in the Suite2p GUI based on mean fluorescence in the red channel after bleed-through correction applied by Suite2p's cell detection algorithm, along with manual correction. For each ROI, Suite2p returned a raw fluorescence timeseries, as well as an estimate of neuropil fluorescence that could contaminate the signal. For each cell, we scaled the neuropil fluorescence by a factor by 0.7 and subtracted this timeseries from the ROI's raw fluorescence timeseries to obtain a neuropil-corrected fluorescence signal for each selected cell.

### Δ*F*/*F* and deconvolution

Once the neuropil-corrected fluorescence was obtained for each neuron, we calculated Δ*F*/*F* for each cell in each imaging frame by calculating (*F* − *F*_baseline_)/*F*_baseline_ for each frame, where *F* is the fluorescence of a given cell at that frame and *F*_baseline_ is the eighth percentile of that cell's fluorescence spanning 450 frames before and after (∼15 s each way, 30 s total). Δ*F*/*F* timeseries were then deconvolved to estimate the relative spike rate in each imaging frame using the OASIS toolbox (Friedrich et al., 2017). We used the AR1 FOOPSI algorithm and allowed the toolbox to optimize the convolution kernel, baseline fluorescence, and noise distribution. A threshold of 0.05 a.u. was applied to remove all events with low magnitude from deconvolved activity timeseries. All analyses were performed with both Δ*F*/*F* and deconvolved activity and showed the same trends. Results in the figures are based on Δ*F*/*F*.

### Pairwise noise correlations

Before computing pairwise noise correlations, each neuron's trial-averaged response to a given target was subtracted from single trial responses in the 333 ms after the target stimulation onset. These values were then correlated between each target neuron and other neuron in the population, but only during trials that the particular target neuron was not stimulated. The goal was to obtain the pairwise correlations of the neurons outside of times that either neuron was stimulated, to measure their natural cofluctuations in activity.

### Influence calculation

Influence of each target on each other neuron was calculated using deconvolved spike rates as well as d*F*/*F*, with similar results. The mean prestimulus response of each neuron (binned across 10 imaging frames, or 333 ms, prior to the target's stimulation) was subtracted from its mean poststimulus response (in the 10 frames, or 333 ms, immediately following the target's stimulation onset). These time windows were chosen to limit the noise in the baseline measurement and to capture the majority of each neuron's response to the stimulation. This value was then normalized by the standard deviation of this difference across all trials. To relate influence level to running speed, the prestimulus running speed was binned into quartiles for each imaging session separately. Influence was then compared across running speed quartiles.

### Histology

After all imaging sessions had been acquired, each mouse was transcardially perfused with saline and then 4% paraformaldehyde. The brain was extracted, cryoprotected, embedded, frozen, and sliced. Once slide mounted, we stained brains with DAPI to be able to identify anatomical landmarks. We used anatomical structure to verify the locations of the viral injections in AC and PPC.

### Experimental design and statistical analysis

Unless otherwise stated, pairwise comparisons were done with two-sided paired or unpaired permutation (i.e., randomization) tests with 10,000 iterations, where *p* = 0.0001 indicates the highest significance achievable given the number of runs performed. All permutation tests were performed for differences in means. Permutation tests were chosen to avoid making unnecessary assumptions about the normality of the data. When multiple comparisons were made between groups, *p* values were Bonferroni-corrected to assess significance. To compare the effects of brain region, cell type, and intersomatic distance on influence, we performed a three-way ANOVA. Post hoc tests were calculated as multiple comparisons using the Tukey–Kramer test. Full values and statistical details from all figures are thoroughly summarized in Tables 1 and 2 and Extended Data Table 2-1.

**Table 1.** Related to Figure 2

		Mean distance to target	Standard deviation	*n*
1	AC all Non-SOM	226.3	110.9	125,496
2	AC positively influenced Non-SOM	162.7	123.4	9,690
3	AC negatively influenced Non-SOM	202.7	109	2,321
4	AC un-influenced Non-SOM	232.3	108	113,485
5	PPC all Non-SOM	209	103.9	171,692
6	PPC positively influenced Non-SOM	159.8	120.1	17,120
7	PPC negatively influenced Non-SOM	170.4	95.5	2,776
8	PPC un-influenced Non-SOM	215.2	100.4	151,796
9	AC all SOM	210	109.9	6,763
10	AC positively influenced SOM	136.3	109.6	532
11	AC negatively influenced SOM	197.9	101.8	137
12	AC un-influenced SOM	219.3	106	6,094
13	PPC all SOM	209	105.4	12,038
14	PPC positively influenced SOM	154.8	113.9	1,245
15	PPC negatively influenced SOM	187.5	103.6	170
16	PPC un-influenced SOM	217.7	101	10,623
Comparison^a^	*p*	Difference	Effect size	
9,13	0.3526	1.50	0.0142	
10,14	7.99 × 10^−4^	−19.50	−0.1734	
11,15	0.4203	9.50	0.0924	
1,5	9.99 × 10^−4^	−17.40	−0.1625	
2,6	9.99 × 10^−4^	−14.58	0.1202	
3,7	9.99 × 10^−4^	14.75	−0.1447	
1,2	9.99 × 10^−4^	63.70	0.5695	
1,3	9.99 × 10^−4^	23.70	0.2137	
1,4	9.99 × 10^−4^	−5.90	−0.0541	
2,3	9.99 × 10^−4^	−40.00	−0.3311	
3,4	9.99 × 10^−4^	−29.60	−0.2742	
5,6	9.99 × 10^−4^	49.20	0.4663	
5,7	9.99 × 10^−4^	38.50	0.3713	
5,8	9.99 × 10^−4^	−6.20	−0.0611	
6,7	9.99 × 10^−4^	−10.70	−0.0911	
7,8	9.99 × 10^−4^	−44.80	−0.4465	
1,9	9.99 × 10^−4^	14.00	0.1269	
2,10	0.0180	12.9.0	0.1051	
3,11	0.3566	−8.74	−0.0804	
9,10	9.99 × 10^−4^	76.00	0.7003	
9,11	0.1229	14.4	0.1328	
9,12	2.00 × 10^−4^	−7.00	−6.49 × 10^−2^	
10,11	1.00 × 10^−4^	−61.6	−5.71 × 10^−1^	
11,12	0.0224	−21.40	−2.02 × 10^−1^	
5,13	0.0699	−1.80	−1.75 × 10^−2^	
6,14	0.0210	7.96	0.0664	
7,15	0.0789	−14.00	−0.1458	
13,14	1.00 × 10^−4^	56	0.532	
13,15	0.0042	23.3	0.224	
13,16	1.00 × 10^−4^	−6.9	−0.0675	
14,15	9.00 × 10^−4^	−32.6	−0.2893	
15,16	3.00 × 10^−4^	−30.3	−0.2997	

a Permutation test.

**Table 2.** Related to Figure 8

Pearson's *r* between noise correlation and influence	AC neurons < 125 µm to target		AC neurons > 125 µm to target		PPC neurons <125 µm to target		PPC neurons >125 µm to target	
	Non-SOM	SOM	Non-SOM	SOM	Non-SOM	SOM	Non-SOM	SOM
*r*	0.1715	0.0215	0.0117	0.0761	0.1489	0.0339	0.0122	0.0511
*p*	8.17 × 10^−96^	0.5289	0.0094	1.42 × 10^−4^	5.43 × 10^−112^	0.1854	0.0021	4.66 × 10^−4^
Number of pairs	14,446	858	48,875	2,493	22,545	1,530	62,910	4,683

### Data and code availability

Processed, trial-aligned neural activity is available at doi.10.17632/xyf46kby2v.1. Raw imaging files are available from the corresponding author upon reasonable request. Code is available at https://github.com/RunyanLab/single_cell_influence_mapping/releases/tag/v1.0.0.

## Results

### Mapping the influence of single excitatory neurons in vivo

**Figure 1.**
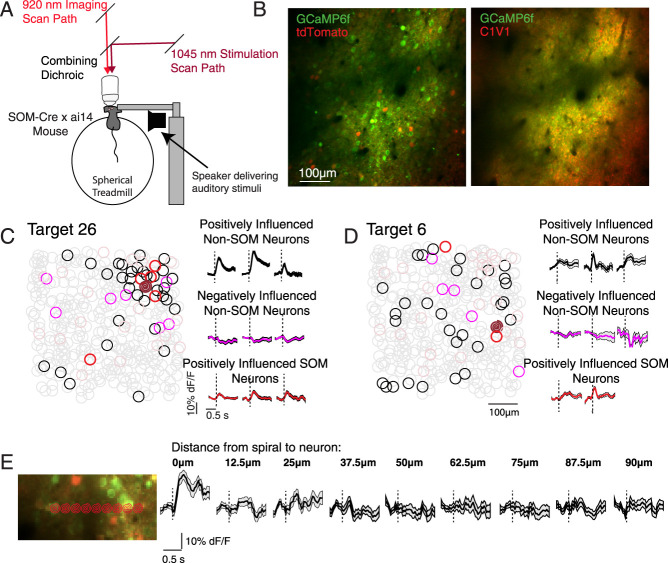
Mapping the influence of single E neurons in vivo. ***A***, SOM-Cre × ai14 mice ran voluntarily on a spherical treadmill throughout experiments. A speaker was used to present pure tones to characterize auditory responses. Excitation for imaging was provided by a laser tuned to 920 nm. Excitation for stimulation was provided by a 1,045 nm laser that was controlled by an independent scan path. ***B***, An excitatory opsin (C1V1) was virally expressed in excitatory neurons. tdTomato was expressed transgenically in SOM neurons, and GCaMP was expressed in all neurons virally, in SOM-Cre x ai14 mice. Left, Example field of view imaged at 920 nm, visualizing GCaMP (green) and tdTomato (red). Right, Same field of view, imaged at 780 nm, where the mScarlet (red, also expressed with C1V1) but not tdTomato can be visualized. ***C***, Left, Example influence map obtained from the field of view in ***B***. Excited neuron's location indicated by the red “spiral.” Significantly positively influenced neurons are circled in black, negatively influenced neurons in magenta. Neurons that were not significantly influenced by the target's stimulation are circled in gray. Right, Example trial-averaged responses of significantly positively influenced E cells, negatively influenced E cells, and positively influenced SOM cells when this neuron was stimulated. ***D***, As in ***C***, for another neuron that was targeted in the same session and field of view. ***E***, Control experiment to determine the spatial resolution of the photostimulations. A neuron was stimulated and then spirals were targeted to adjacent locations, as indicated by the red spirals superimposed on the image. Right, Trial-averaged responses of the neuron during spiral scans that were directed to each location indicated on the left. The neuron was only significantly responsive when the spiral was focused directly on it. We only included other neurons that were at least 25 µm away from the spiral in our influence measurements. According to our tests, this is a conservative enough approach because we rarely observed direct stimulation of a neuron even 12.5 µm away. Shading: SEM.

Our goal was to compare the functional structure of recurrent networks in layer 2/3 of the auditory and parietal cortex, where population activity dynamics differ dramatically across areas (Runyan et al., 2017; Valente et al., 2021; Khoury et al., 2022). We expressed a red shifted opsin (C1V1) in excitatory (E) neurons, a red fluorophore (tdTomato) in SOM neurons, and a green calcium indicator (GCaMP6f) in all neurons in both the auditory cortex (AC) and posterior parietal cortex (PPC) of 10 SOM-Cre × ai14 mice (Fig. 1*A*,*B*). During imaging sessions (25 AC sessions and 30 PPC sessions, AC and PPC were imaged within the same mice on alternating days), mice ran voluntarily on a spherical treadmill. To map the influence of E neurons on local SOM and Non-SOM neurons in AC and PPC, we performed spiral scans with a 1,045 nm laser beam over individual C1V1-expressing E neurons to trigger action potentials (Rickgauer and Tank, 2009; Chettih and Harvey, 2019) while simultaneously raster scanning a 920 nm laser beam to monitor spike-related calcium activity in GCaMP-expressing neurons. In a typical imaging session, we targeted spirals (100 ms in duration) to ∼30 individual E neurons for at least 100 trial repeats, in pseudorandom order, with 1 s between stimulations. Successfully stimulated neurons were, by definition, excitatory neurons, as they expressed C1V1 under the CaMKII promoter. The other neurons in the field of view were sorted based on their tdTomato expression: tdTomato+ neurons were considered SOM, and tdTomato− neurons were considered Non-SOM, which can include Non-SOM inhibitory interneurons, but the majority would be excitatory neurons. Each field of view contained 14.8 ± 6.6 SOM neurons (mean ± standard deviation, here and onward) and 222.9 ± 96.0 Non-SOM neurons in AC and PPC. Of the 30.6 ± 7.4 neurons targeted for photostimulation, 22.6 ± 6.9 were successfully stimulated. All imaging and stimulations were targeted to similar depths from the cortical surface (120–300 µm).

The “influence” of each successfully stimulated neuron on every other simultaneously imaged neuron was then assessed. We defined “influence” of a photostimulation target on another neuron as the other neuron's mean response to the target's stimulation (response during 1 s after the target's stimulation onset minus response in the ∼300 ms before stimulation onset) across all trials, divided by its standard deviation. We successfully stimulated 506 E neurons in AC, testing 125,496 potential influences on Non-SOM and 6,846 potential influences on SOM neurons. In PPC, we successfully stimulated 577 E neurons, testing 171,692 potential influences on Non-SOM neurons and 12,152 potential influences on SOM neurons in PPC. On average, a sparse subset of neighboring neurons were positively influenced (7.9% ± 8.7% of all neurons, defined as influence greater than 99% of trial shuffled data, see Materials and Methods, considering only neurons at least 25 µm away from the target), and a smaller subset of local neurons were negatively influenced (3.4% ± 3.5% of all neurons, defined as influence less than 99% of trial shuffled data; Fig. 1*C*,*D*).

To estimate the spatial specificity of the spiral stimulation, in most sessions we selected a single C1V1-expressing neuron that was on the edge of the viral injection site. We then systematically stimulated the location of the neuron's soma, and neighboring distances at 12.5 µm increments (Fig. 1*E*). For most targets, significant responses were only evoked when the spirals were targeted within 12.5 µm of the soma. To be conservative, in subsequent analyses, neurons within 25 µm of the stimulation target were excluded.

### Center/surround organization of local influence of single E neurons

**Figure 2.**
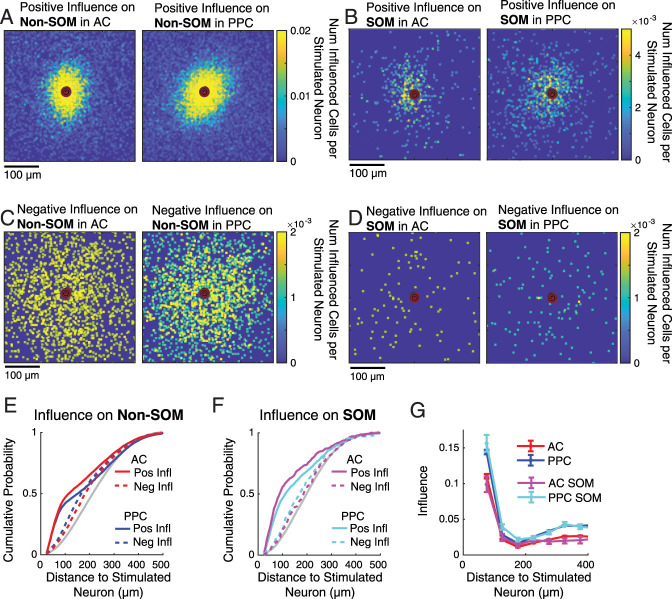
The spatial spread of excitatory influence in AC and PPC. ***A***, Maps of influence on Non-SOM neurons, across all targeted E neurons and all fields of view in AC (left) and PPC (right). Each targeted neuron was centered, and the pixel locations of its significantly positively influenced neurons set to 1. Then, all 506 AC maps and 577 PPC maps were summed and divided by the total number of targeted neurons, so that the color at each pixel location indicates the number of significantly positively influenced Non-SOM neurons per targeted neuron, at each relative location. ***B***, As in ***A*** but for positive influence on SOM neurons (*n*'s of targeted neurons are the same as in ***A***). ***C***, ***D***, As in ***A*** and ***B*** but for negative influence. ***E***, Cumulative distributions of the intersomatic distances between the targets and significantly influenced Non-SOM cells, in AC (red) and PPC (blue). Gray, distributions of distances of all Non-SOM neurons to the target. Solid, positively influenced neurons; dashed, negatively influenced neurons. See Table 1 for *n*'s. ***F***, As in ***E***, but showing the distributions of distances of SOM neurons to the targets. ***G***, Same data as in ***E*** and ***F***, but plotting the mean influence on all neurons, binned by distance to the target. In all panels, only neurons at least 25 µm from the target were considered. See Table 1 and Extended Data Table 2-1 for full values and statistics.

To display and analyze the spatial spread of E neurons' local network influence in each area, we centered each targeted neuron and overlaid and summed the relative spatial positions of significantly positively or negatively influenced neurons, normalizing by the total number of targeted neurons (Fig. 2*A–D*). Visually, the spatial extent of positive influence was restricted and centered around the stimulated neuron in both cortical regions, though this zone of positive influence appeared wider in PPC than in AC (Fig. 2*A*,*B*). In contrast, the spatial pattern of negative influence was more wide and diffuse than positive influence and appeared more dominant in AC than PPC (Fig. 2*C*,*D*).

10.1523/JNEUROSCI.1385-24.2025.t2-1 Table 2-1 ANOVA and Posthoc Tests, Related to Figure 2. Download Table 2-1, DOCX file.

To quantify the differences in the spatial spread of positive and negative influence of E cells in AC and PPC, we measured the intersomatic distance from each targeted neuron to every other neuron in the field of view. We then compared the distributions of intersomatic distances of significantly positively and negatively influenced Non-SOM (Fig. 2*E*) and SOM (Fig. 2*F*) neurons. In both regions, positively influenced neurons tended to be closer to the targeted neurons than both negatively influenced and the full distribution of imaged neurons (AC Non-SOM: positively influenced vs negatively influenced *p* = 0.000999; PPC Non-SOM: *p* = 0.000999; AC SOM: *p* = 0.000999; PPC SOM: *p* = 0.0001; see Table 1 for full values and statistics).

To test our hypothesis that recurrent excitation spreads more widely in PPC than in AC, we then compared the distribution of distances to positively influenced neurons across areas. Consistent with our hypothesis, the zone of positively influenced neurons was more spatially restricted in AC than in PPC: the distances from positively influenced cells to the targeted neurons were longer in PPC than AC (Fig. 2*A*,*E*,*G*; *p* = 0.00099). Similarly, distances to positively influenced SOM neurons were longer in PPC than in AC (Fig. 2*B*,*F*,*G*; *p* = 0.00070). However, the distance to negatively influenced Non-SOM neurons was longer in AC than PPC (Fig. 2*C*; *p* = 0.000999). The distance to negatively influenced SOM neurons did not differ in AC and PPC (*p* = 0.4203).

To simultaneously test for effects of brain region, cell type, and intersomatic distance on the targets' influence, we binned the intersomatic distances from targets to other neurons and performed a three-way ANOVA (Fig. 2*G*; results reported in Extended Data Table 2-1). There were significant main effects of brain area and intersomatic distance (*p* < 0.0001), and significant interactions between brain area and intersomatic distance (*p* < 0.0001) and between cell type and distance (*p* = 0.0072). Post hoc tests revealed greater influence on both SOM and Non-SOM neurons in PPC compared with AC neurons at distances <100 µm (*p* = 9.59 × 10^−6^; Tukey–Kramer test; for multiple comparisons, see Extended Data Table 2-2 for full list of statistical comparisons).

To summarize, stimulation of E cells resulted in a central zone of positive influence on the local population, with a diffuse “surround” zone of negative influence. Positive influence spread farther in PPC, but negative influence spread farther in AC.

### The effects of single E neuron stimulation on local population activity

**Figure 3.**
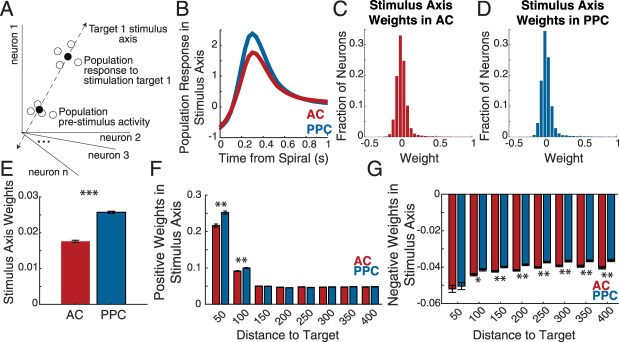
Excitatory neuron influence on the local population. ***A***, A “stimulus axis” was defined in high dimensional neural activity space for the population's response to each specific target's stimulation that best aligned with the population's response to the target stimulation. ***B***, The population mean response in the stimulus axis (defined independently for each target stimulus) is plotted for AC and PPC. *n* = 506 AC populations and 577 PPC populations. ***C***, The distribution of weights along the stimulus axis, of all AC neurons at least 25 µm from the targets (*n* = 132,259 neurons). ***D***, As in ***B***, for PPC neurons (*n* = 183,730 neurons). ***E***, Same data as in ***B*** and ***C***, but plotted as bar plots of the mean stimulus axis weight across all AC and all PPC neurons. Error bars indicate standard error of the mean. PPC weights were significantly more positive than AC weights (*p* = 1.00 × 10^−4^, permutation test). ***F***, ***G***, The positively (***F***) and negatively (***G***) weighted neurons in each stimulus axis were separated and binned by distance to the stimulated neuron in AC and PPC. Asterisks indicate Bonferroni-corrected significance in unpaired permutation tests comparing the weights between AC and PPC in each distance bin. ***p < 0.001, **p < 0.01, *p < 0.05.

Our previous analysis focused on the effects of target stimulation on other individual neurons. To capture the population-level impact of the targeted neuron's stimulation, we next studied the population pattern of local influence in “population activity space.” We reasoned that if the local recurrent excitatory synapses are denser and connect neurons over wider distances in PPC, this should also be evident in the pattern of neurons in each local population that is positively versus negatively affected by the target neuron's activity. In high dimensional space, where each axis is defined as the activity of one neuron in the population (*n* neurons in a population define an *n*-dimensional space), we identified a “stimulus axis” for each stimulated neuron (Fig. 3*A*). This is the axis in *n*-dimensional space that connects the population's average prestimulus activity to the population's response to the stimulation of that neuron. When we projected population activity onto this axis, using both SOM and Non-SOM neurons, a robust stimulus-evoked population response was revealed (Fig. 3*B*). Next, we examined the distribution of weights of the nonstimulated neurons that defined each “stimulus axis.” The mean stimulus axis weight was more positive in PPC than AC (AC: 0.018  ±  0.080, PPC: 0.026  ±  0.089, *p* = 0.0001, permutation test), suggesting that while the density of E influence in the two regions is sparse, influence on the local population was more positive in PPC than in AC (Fig. *3C–E*).

To consider the relationship between stimulus axis weight and distance to the stimulated target neuron, we next split all nontargeted neurons into positively (Fig. 3*F*) and negatively weighted subsets (Fig. 3*G*), binned by their intersomatic distances to the target, and compared the magnitude of influence in each spatial bin between AC and PPC. The magnitude of positive weights was higher in PPC than that in AC for spatial bins 50 and 100 µm from the target (Fig. 3*F*; 50 µm *p* = 0.0001; 100 µm *p* = 0.0001; 150 µm *p* = 0.4323; 200 µm *p* = 0.0315; 250 µm *p* = 0.0085; 300 µm *p* = 0.3221; 350 µm *p* = 0.2590; 400 µm *p* = 0.4143). When we compared the magnitudes of negative weights by distance bin, a complementary pattern emerged. At distances greater than or equal to 100 µm, the magnitude of negative influence was greater in AC than in PPC (Fig. 3*G*; 50 µm *p* = 0.5730; 100 µm *p* = 0.0015; 150–325 µm *p* = 0.0001; 350 µm *p* = 0.0002; 400 µm *p* = 0.0001). Bonferroni-corrected significance for these analyses is indicated in Figure 3*F*,*G*.

To summarize, we characterized the spatial distributions of positively and negatively weighted neurons in the population response to each target's stimulation. The results were consistent with the single neuron analyses in Figure 2, as positive weights were larger in magnitude overall in PPC than in AC, and negative weights were larger in magnitude in AC than in PPC, especially for neurons at farther distances from the targeted neuron.

### Influence and behavioral state

While the mice in our study were not performing a task, they were allowed to run voluntarily on the spherical treadmill throughout the imaging sessions. In this context, mice tend to shift between low arousal states with little movement and high arousal states with running (McGinley et al., 2015; Vinck et al., 2015; Khoury et al., 2023). In AC, the relationship between neural activity and running speed is heterogeneous, with positive and negative modulation of activity across neuron types (Bigelow et al., 2019; Yavorska and Wehr, 2021; Khoury et al., 2023). In PPC, on the other hand, activity is positively modulated by running, especially in SOM neurons (Khoury et al., 2023). Given the different relationships between running behavior and activity in AC and PPC, we reasoned that the local influence of E neurons could be differentially linked to behavioral state in the two areas. Specifically, we expected that the stronger running modulation of population activity in PPC could lead to different network states with stronger or weaker local recurrence.

**Figure 4.**
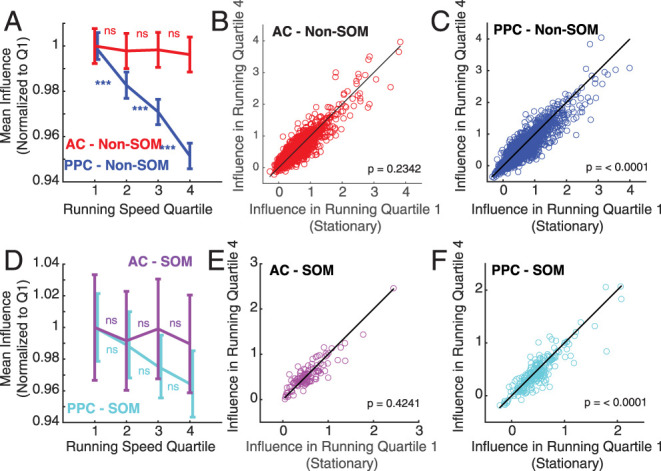
Excitatory influence depends on running behavior in PPC but not AC. ***A***, Mean positive influence in each running quartile in AC and PPC non-SOM neurons. Statistical comparisons are pairwise tests between neighboring bins. ***B***, Influence on AC Non-SOM neurons in running quartile 1 (stationary) plotted against influence on same neurons in running quartile 4 (highest speed). *n* = 3,058 significantly positively influenced AC Non-SOM neurons. Black line, *x* = *y*, for ease of comparison across running quartiles. ***C***, Influence on PPC Non-SOM neurons in running quartile 1 (stationary) plotted against influence on same neurons in running quartile 4 (highest speed). *n* = 4,566 significantly positively influenced PPC Non-SOM neurons. Black line, *x* = *y*. ***D–F***, As in ***A–C***, for SOM neurons. *n* = 140 significantly positively influenced SOM AC neurons and 255 SOM PPC neurons. In all panels, *** indicates *p* < 0.001, with Bonferroni’s correction.

To test the hypothesis that running behavior modulates local recurrent excitatory interactions more strongly in PPC, we first measured the mean running speed during the 20 imaging frames (660 ms) preceding each photostimulation. We then split the distribution of prestimulus running speeds into quartiles, for each imaging session separately. The first quartile corresponded to mice being stationary on the treadmill. We then measured the mean influence of E neuron stimulation on other neurons in each running speed quartile, restricting this analysis to include only neurons that were significantly positively influenced when considering all trials and running speeds. The magnitude of positive influence on Non-SOM neurons was not affected by running speed in AC (influence in running speed quartiles 1 vs 2: *p* = 0.4439; 2 vs 3: *p* = 0.8644; 3 vs 4: *p* = 0.4730; 1 vs 4: 0.2342; Fig. 4*A*). However, the magnitude of positive influence on Non-SOM in PPC decreased systematically with running speed (influence in running speed quartiles 1 vs 2, 2 vs 3, 3 vs 4 and 1 vs 4: *p* < 0.0001; Fig. 4*A*). To examine the full distribution of running-related effects on influence, we then plotted the influence on every Non-SOM neuron in running speed quartiles 1 (stationary) vs 4 (highest running speed; Fig. 4*B*,*C*). In AC, influence was equally likely to be larger and smaller with higher running speed. In PPC, on the other hand, more neurons were more strongly influenced in the stationary running quartile than with high speed running (Fig. 4*C*). We repeated this analysis to compare positive influence on SOM neurons with running (Fig. 4*D–F*), with similar though weaker trends. Influence on SOM neurons was unrelated to running speed in AC (Influence in running quartiles 1 vs 2: *p* = 0.4599; 2 vs 3: *p* = 0.5411; 3 vs 4: 0.4457; 1 vs 4: 0.4241). Positive influence on SOM neurons in PPC was strongest while mice were stationary (influence in running quartiles 1 vs 2: *p* = 0.1296; 2 vs 3: 0.0908; 3 vs 4: 0.2632; 1 vs 4: *p* < 0.0001).

**Figure 5.**
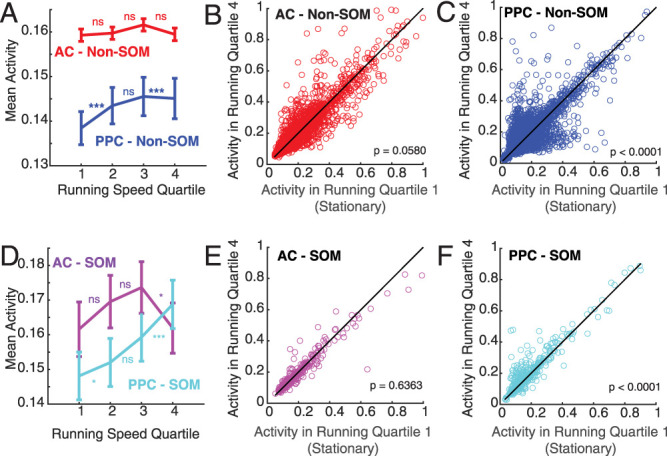
Activity increases in PPC SOM and Non-SOM neurons with running. ***A***, Mean activity (d*F*/*F*) in each running quartile in AC and PPC non-SOM neurons. Statistical comparisons are pairwise tests between neighboring bins. ***B***, Mean activity of AC Non-SOM neurons in running quartile 1 (stationary) plotted against influence on same neurons in running quartile 4 (highest speed). *n* = 5,990 AC Non-SOM neurons. Black line, *x* = *y*, for ease of comparison across running quartiles. ***C***, Mean activity of PPC Non-SOM neurons in running quartile 1 (stationary) plotted against influence on same neurons in running quartile 4 (highest speed). *n* = 5,259 significantly positively influenced PPC Non-SOM neurons. Black line: *x* = *y*. ***D–F***, As in ***A–C***, for SOM neurons. *n* = 317 SOM AC neurons and 384 SOM PPC neurons. In all panels, *** indicates *p* < 0.001, with Bonferroni’s correction.

We next compared the average activity of neurons across the levels of running speed, to test whether our results replicate previous findings showing more positive running-related modulation of activity in PPC than in AC (Khoury et al., 2023). Overall, Non-SOM activity increased with running speed in PPC, but not AC (Fig. 5*A–C*). However, running effects on activity were heterogeneous across Non-SOM neurons; a large number of Non-SOM neurons in PPC did have higher activity in the stationary trials than in high speed running (neurons below the unity line in Fig. 5*C*). The positive relationship between activity and running speed was more uniform in SOM neurons than in Non-SOM neurons in PPC. Activity in most PPC SOM neurons was positively modulated with running (Fig. 5*D*,*F*). Together, the results demonstrate that the relationship between running speed and activity is generally weak and mixed in AC, but strong and positive in PPC, especially in SOM neurons, replicating previous findings (Khoury et al., 2023).

In summary, in AC, activity of Non-SOM and SOM neurons was not consistently affected by running speed, nor was the influence of E neurons on them. In contrast, increases in running speed led to increases in activity especially in SOM neurons in PPC, while the influence of E neurons in PPC decreased with running speed.

### Influence and pairwise noise correlations

**Figure 6.**
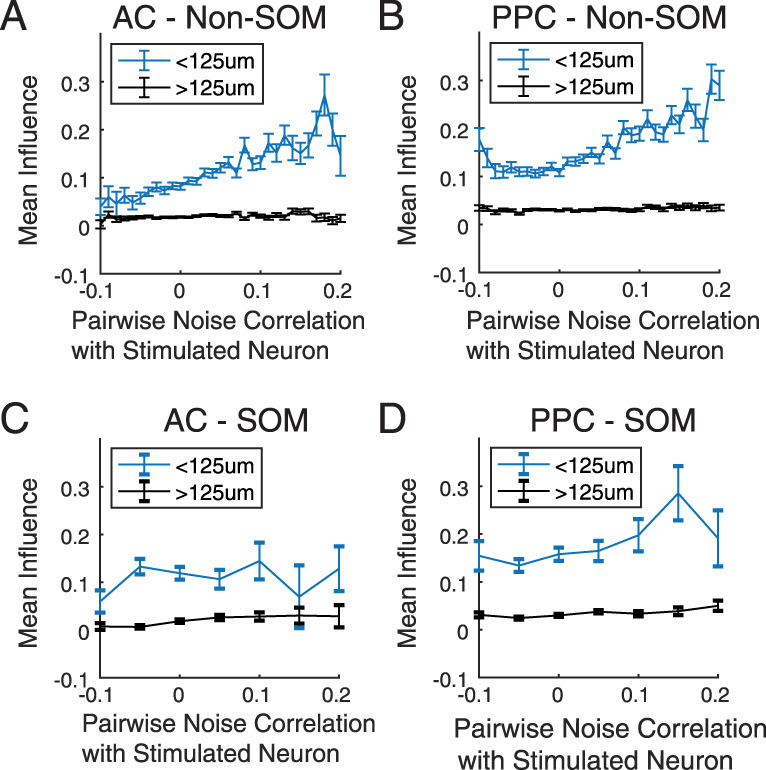
The relationship between excitatory influence and pairwise noise correlation. ***A***, Pairwise Pearson’s correlations were computed between each target–neuron pair and binned and related to the target's influence on the other neuron. Noise correlation and influence were more positively correlated for Non-SOM neurons within 125 µm of the target (blue), than for neurons farther away (black). See Table 2 for full values and statistics. ***B***, As in ***A***, for PPC Non-SOM neurons. As in AC, neighboring Non-SOM with stronger noise correlations tended to be more positively influenced. ***C***, As in ***A***, for AC SOM neurons. Noise correlations and influence were not positively related in neighboring SOM neurons but were weakly correlated in SOM neurons at greater distances. ***D***, As in ***A–C***, for PPC SOM neurons.

Pairwise noise correlations have often been used to assess local network state (Cohen and Kohn, 2011). Positive pairwise noise correlations can be produced by a combination of common synaptic input and direct monosynaptic interconnections between the two neurons, while positive influence requires some synaptic path from the stimulated neuron to the influenced neuron. In the visual cortex, several lines of experimental evidence demonstrate that positive pairwise noise correlations could arise at least in part from direct monosynaptic connections between the two neurons: closely neighboring neurons with positive noise correlations (greater shared variability) have a higher probability of forming monosynaptic connections (Ko et al., 2011). These neurons also have greater positive influence on each other, in similar conditions as in the experiments in this study (Chettih and Harvey, 2019). In PPC, pairwise noise correlations are more positive and higher in magnitude than in the sensory cortex (Runyan et al., 2017; Khoury et al., 2022), but it is unclear if this reflects an underlying increase in local excitatory connections compared with the sensory cortex. To test if positive noise correlations are also related to influence between neurons in AC and PPC, we computed Pearson's correlation coefficients between the trial mean-subtracted activity traces of every target–neuron pair, using only imaging frames outside of the target neuron's stimulation trials, and related this *r* value to the influence of the target on the other neuron. In both AC and PPC, influence on Non-SOM neurons was positively related to pairwise noise correlation for cell pairs with intersomatic distances within 125 µm (Pearson’s *r* between influence and pairwise noise correlation between neuron and target: A1 Non-SOM: *r* = 0.17, *p* < 0.000001; PPC Non-SOM: *r* = 0.15, *p* < 0.000001; Fig. 6*A*,*B*). The slope of this relationship was closer to zero for pairs with intersomatic distances >125 µm, which is consistent with results in mouse V1 (Chettih and Harvey, 2019; A1 Non-SOM: *r* = 0.01, *p* = 0.0094; PPC Non-SOM: *r* = 0.01, *p* = 0.0021). In contrast, the relationship between pairwise noise correlation and influence on SOM neurons was not different from zero among neurons near the target in AC and PPC (Fig. 6*C*; AC SOM within 125 µm of the target: *r* = 0.02, *p* = 0.53; PPC SOM: *r* = 0.03, *p* = 0.18). At farther distances, noise correlations and influence on SOM neurons did have a weakly positive relationship in AC and PPC (AC >125 µm from the target: *r* = 0.08, *p* = 0.00014; PPC: *r* = 0.05, *p* = 0.00046; Fig. 6*C*,*D*; Table 2 for full values).

**Figure 7.**
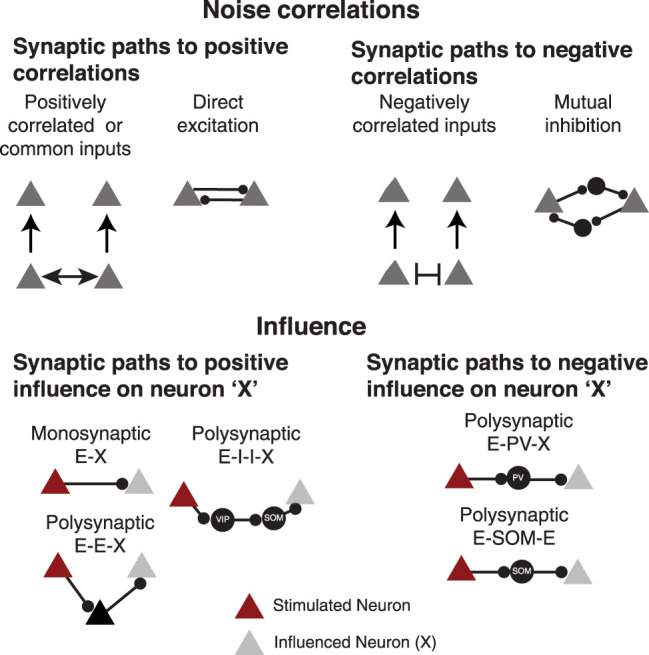
Synaptic mechanisms underlying noise correlations and influence. Top, Multiple synaptic paths can explain positive and negative pairwise noise correlations. While some solutions include direct synaptic connections between the correlated neurons, most include additional, likely unobserved neurons. Bottom, Multiple synaptic paths from the stimulated neuron (crimson) to the influenced neurons (gray) exist. While not all include a monosynaptic connection from the stimulated neuron to the influenced neuron, there is a causal flow of activity from the stimulated neuron to the influenced neurons.

Noise correlations and influence are correlated in the visual cortex, as neurons with strong and positive pairwise noise correlations have a higher probability of forming monosynaptic excitatory connections (Ko et al., 2011). Yet we found that PPC Non-SOM neurons with negative pairwise correlations can also positively influence each other (Fig. 6*B*). It is thus important to iterate that pairwise noise correlations can arise from many potential underlying connectivity schemes, many of which do not include causal flow of activity between the two neurons (Fig. 7, top). Positive and negative influence requires some causal chain of connections from the stimulated neuron to the influence neuron, though the precise synaptic connectivity pattern producing the influence cannot be determined with our method (Fig. 7, bottom). The intermediary neurons (Fig. 7, bottom, black triangle) may be unobserved neurons in other layers or brain regions.

### Influence and sound frequency tuning in AC

In V1, E neurons with similar feature tuning have higher monosynaptic connection probabilities (Ko et al., 2011), which would in theory allow recurrent E connections to provide signal amplification among similarly tuned neurons. However, in vivo single cell-resolution stimulation does not always have this predicted signal amplifying effect. Single neuron stimulation in V1 in vivo can lead to polysynaptic inhibitory influence on neurons with tuning similar to the stimulated neuron (Chettih and Harvey, 2019), a potential mechanism for feature competition. Yet stimulation of ensembles of similarly tuned neurons leads to more excitatory influence on other, similarly tuned neurons (Oldenburg et al., 2024). To determine whether activation of single neurons in mouse auditory cortex triggers positive or negative influence on similarly tuned neurons, we played pure tone stimuli to characterize the frequency tuning preference for each sound-responsive neuron in AC. We then related neurons' pure tone frequency preferences to their influence. Because sensory responses in PPC are limited outside of a task context (Pho et al., 2018), we did not measure frequency tuning in PPC.

**Figure 8.**
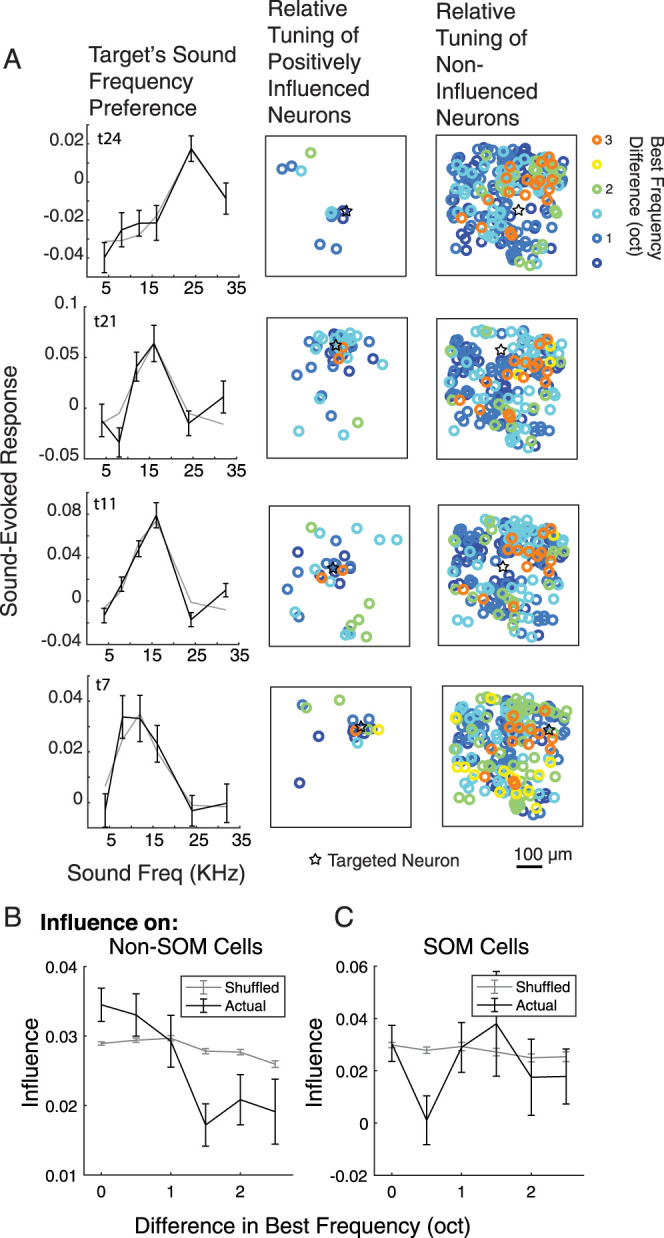
The relationship between excitatory influence and sound frequency preference. ***A***, Left, For each sound-responsive and frequency-selective target, we measured its sound frequency preference. We then measured the difference in preferred frequency between the target and all positively influenced (middle) and noninfluenced (right) neurons. Each row shows an example sound responsive target and relative tuning and locations of its influenced and noninfluenced neurons in the field of view, all from the same imaging session. Target locations are indicated with black stars in the influence maps. ***B***, Mean influence binned by difference in best frequency between the target and Non-SOM neurons (black). For comparison to an unorganized influence map, the sound frequency preferences were randomly shuffled among the neurons before comparing influence to relative best frequency (gray). The correlation between influence and relative best frequency was significantly more negative in the actual than shuffled data (*p* = 0.014). ***C***, As in ***B***, for SOM neurons. The correlation between influence and relative best frequency was not significantly different in the actual and shuffled data (*p* = 0.12).

For this analysis, we considered only AC neurons that were sound responsive and had a preferred sound frequency (71,450 target–neuron pairs; Fig. 8*A*). We estimated each neuron's preferred sound frequency (best frequency, BF) by using the best fit Gaussian to the neuron's trial-averaged sound-evoked responses and then measured the difference in BF between each stimulated target and each other neuron in the field of view, in octaves (Fig. 8*A*). Next, we related the target's influence on the neuron to their BF difference. At a single binned frequency difference (1 oct), influence on SOM neurons was significantly lower than that on Non-SOM neurons (*p* = 0.0001998). Finally, for SOM and Non-SOM neurons separately, we compared the relationship between influence and BF difference to a shuffled distribution, where we randomly shuffled the frequency preferences across all neurons in the field of view. This allowed us to take into account the actual distribution of BFs in each dataset and cell type when considering the relationship between relative tuning and influence. Influence was stronger on Non-SOM neurons with similar frequency preferences, and weaker on Non-SOM neurons with more different frequency preferences, compared with the shuffled distribution (Pearson's correlation between influence and relative best frequency was significantly more negative than 500 shuffles, *p* = 0.014, *r* = −0.0257; Fig. 8*B*). Influence on SOM neurons, however, was similar to the shuffled distribution and did not relate to relative tuning (*p* = 0.12, *r* = 0.05; Fig. 8*C*).

## Discussion

**Figure 9.**
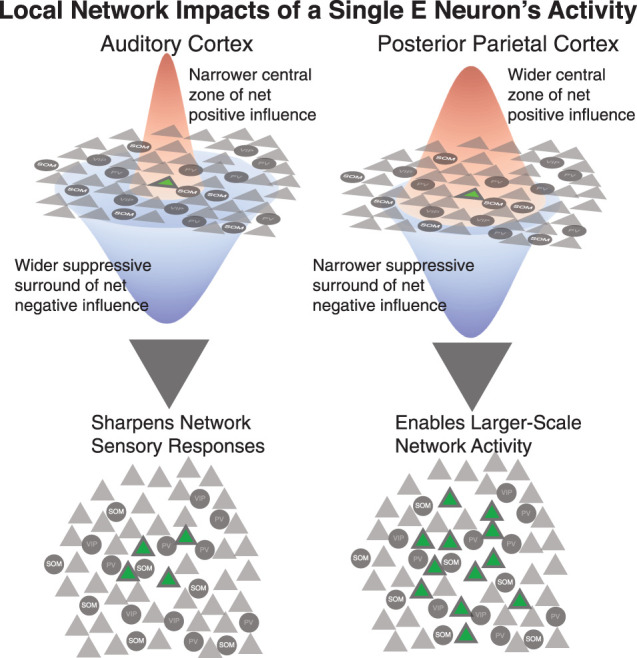
Differently balanced E/I networks in AC and PPC. Top, Single neuron stimulation triggers positive changes in activity (influence) in other neurons located within a narrow zone centered on the stimulated neuron, and negative changes in activity in a more diffuse, spatially spread “surround” region. These two zones, depicted in red and blue, are the net result of the pattern of E-E, E-I-E, and other polysynaptic connections from the stimulated cell to the influenced neurons (Fig. 7). In AC and PPC, these central positive and suppressive surround zones have different relative scales. In AC, the positive zone is more restricted and the surround zone is wider. In PPC, the positive and suppressive zones are more closely matched in spatial scale. These differences could be driven by different densities and connection patterns of excitatory synapses and inhibitory synapses. Bottom, These differently balanced E/I subnetworks produce different local network dynamics and coding regimes. Left, In AC, excitation remains more restricted, allowing for more fine-scale sensory encoding. Right, In PPC, excitation can spread more easily across the population, allowing for larger-scale networks to support sensory integration and forming associations between stimuli and behavioral choices.

Population activity dynamics differ systematically across the cortical processing hierarchy (Runyan et al., 2017; Safavi et al., 2018; Khoury et al., 2022). It has been unclear though to what extent these region-specific dynamics are driven by extrinsic inputs with differing dynamics or by differences in intrinsic connectivity patterns across regions that cause the networks to respond to inputs with different dynamics. In the current study, our goal was to determine whether intrinsic differences in recurrent network structure could contribute to setting the properties of local population activity dynamics. We photostimulated individual excitatory (E) neurons and simultaneously monitored spike-related calcium activity in the neighboring population of E and somatostatin-expressing inhibitory interneurons (SOM), within layer 2/3. We found that single E neurons exert a network impact with similar center/surround organization in both AC and PPC, and yet the relative spatial extents of the center and surround regions of influence differed across areas. In AC, a narrow central zone of positive influence was surrounded by a large, diffuse, negative influence zone. In PPC, a wider central zone of positive influence was surrounded by a weaker and smaller zone of negative influence. These differing ratios of network center and surround responses to the activation of single excitatory neurons suggest that recurrent network structure could dictate the activity regimes of the local population. In the auditory cortex, excitatory activity remains sparse, as it is held in check by the strong suppressive surround. The result is neural population activity that is relatively decorrelated in AC. In the parietal cortex, excitation can spread more easily across neurons, with a weaker suppressive surround and/or stronger excitatory recurrence, increasing the correlations in activity across neurons. These differences are conceptualized in Figure 9.

The center-surround pattern of local influence that we observed in AC and PPC is consistent with known organization of excitatory and inhibitory microcircuits in the sensory cortex (Adesnik et al., 2012; Chettih and Harvey, 2019; Oldenburg et al., 2024), providing further support that this is a canonical feature of local circuit organization in the cortex. In sensory cortices, the probability of a monosynaptic connection between two excitatory neurons decays rapidly with intersomatic distance (Perin et al., 2011; Levy and Reyes, 2012; Rossi et al., 2020; Campagnola et al., 2022; Hage et al., 2022). Single neuron stimulation in vivo, which allows for a network-level measurement of a neuron's influence, similarly reveals that the influence of single excitatory neurons decays with anatomical distance across the local population in the visual cortex (Chettih and Harvey, 2019; Oldenburg et al., 2024). The inhibitory “surround” triggered in the population by single E cells has a similar spatial pattern across studies, and SOM interneurons may be a key contributor to this surround organization, as SOM neurons receive lateral excitatory synapses from more spatially distant neurons (Adesnik et al., 2012; Kato et al., 2017). We were surprised to find that influence did not spread over farther distances in SOM neurons than Non-SOM neurons in our study (Fig. 2, Table 1). Perhaps the SOM neurons are more difficult to influence in the context of our experiments, due to differing properties of excitatory synapses onto SOM neurons (Tremblay et al., 2016), or the activity levels of SOM neurons in the spontaneous context of our experiments. Alternatively, the suppressive network surround could be produced through Non-SOM interneurons, such as PV neurons.

Our method, which relies on measuring changes in spike-related calcium activity, cannot reveal the synaptic path from the targeted neuron to the neurons that are positively or negatively influenced (Figs. 7, 9). Positive influence could result from monosynaptic E–E connections or polysynaptic excitatory connections that include at least three neurons. The intermediate E neurons may be local to the region of the stimulated neuron, or unobserved, even distant neurons that mediate an excitatory feedback loop with AC or PPC. Negative influence is necessarily polysynaptic and could be triggered through SOM or parvalbumin-positive (PV) interneurons. Furthermore, the positive and negative influence that we have measured is the net result of the local excitatory and inhibitory synapses that were activated when the targeted neuron was stimulated. For example, a neuron that was positively influenced could receive both excitatory and inhibitory synapses that are downstream of the stimulated neuron, but in the conditions of our experiment, the net result was an increase in the influenced neuron's activity. The presence or absence of specific synaptic connections cannot reliably predict network-level patterns of activity, and so measuring the functional impacts of a neuron's activity is key to understanding its potential impacts on computation (Randi et al., 2023; Pospisil et al., 2024).

Short- and long-term plasticity occur at cortical synapses, and brain state-dependent changes in neuromodulatory release can change synaptic efficacy and baseline activity levels in specific cell types (Fanselow et al., 2008; Chen et al., 2015; Kuchibhotla et al., 2016; Garcia-Junco-Clemente et al., 2019), which would change the strength and pattern of positive and negative influence. We have measured local functional network influence of single E neurons in one specific context, when mice were voluntarily running on the spherical treadmill, in the absence of experimentally controlled sensory stimuli. We suspect that these influence patterns strongly depend on factors like brain state and sensory stimulation. Indeed, under different stimulation parameters and network conditions in the primary visual cortex, the effects of optically stimulating excitatory neurons in vivo can be suppressive (Chettih and Harvey, 2019) or enhancing (Oldenburg et al., 2024). Influence mapping, especially in PPC, should be repeated in task-engaged and other behavioral states in future studies.

Finally, anatomical distance between cell bodies is not the sole determinant of connection probability between two neurons in the cortex. Neurons with similar tuning in V1 have a higher probability of making stronger, bidirectional synaptic connections with each other (Ko et al., 2011; Li et al., 2012; Cossell et al., 2015; Znamenskiy et al., 2024), a connectivity motif that enables amplification of feedforward signals. In PPC, excitatory neurons with opposing choice-tuning disynaptically inhibit each other (Kuan et al., 2024). Our results support the possibility that local connectivity is also functionally specific among E cells in the auditory cortex, as single E neurons positively influenced the activity of neighboring E neurons with similar sound frequency tuning, measured using pure tones (Fig. 8). Influence on SOM neurons, however, was random with respect to frequency tuning, potentially enabling SOM neurons to provide a nonspecific, suppressive modulation of activity. Future studies relating influence and selectivity for more complex auditory features may reveal additional principles of response selectivity and influence. We did not measure sound-related activity in PPC, as sensory stimulus-evoked responses in PPC are weak or even nonexistent in passive contexts (Pho et al., 2018). The relationship between influence and task-related tuning should be examined in PPC in future studies.
