# Supplementary material for: Local Differences in Network Organization in the Auditory and Parietal Cortex, Revealed with Single Neuron Activation
Source: J Neurosci. 2025 Jan 31;45(11):e1385242025. doi: 10.1523/JNEUROSCI.1385-24.2025 (PMC11905346; doi:10.1523/JNEUROSCI.1385-24.2025)
Supplement: Table 2-1 — ANOVA and Posthoc Tests, Related to Figure 2. Download Table 2-1, DOCX file. [file jneuro-45-e1385242025-s001.docx]

**Table 2-1: ANOVA and Posthoc Tests, Related to Figure 2**

| **Group Number** | **Group Name** | **Mean Influence** | **Standard Deviation** |
| --- | --- | --- | --- |
| 1 | AC,Non-SOM,60um | 0.257 | 0.002 |
| 2 | PPC,Non-SOM,60um | 0.343 | 0.002 |
| 3 | AC,SOM,60um | 0.233 | 0.008 |
| 4 | PPC,SOM,60um | 0.335 | 0.007 |
| 5 | AC,Non-SOM,90um | 0.095 | 0.002 |
| 6 | PPC,Non-SOM,90um | 0.126 | 0.001 |
| 7 | AC,SOM,90um | 0.090 | 0.007 |
| 8 | PPC,SOM,90um | 0.142 | 0.005 |
| 9 | AC,Non-SOM,120um | 0.029 | 0.002 |
| 10 | PPC,Non-SOM,120um | 0.038 | 0.001 |
| 11 | AC,SOM,120um | 0.036 | 0.006 |
| 12 | PPC,SOM,120um | 0.050 | 0.005 |
| 13 | AC,Non-SOM,150um | 0.012 | 0.001 |
| 14 | PPC,Non-SOM,150um | 0.015 | 0.001 |
| 15 | AC,SOM,150um | 0.010 | 0.006 |
| 16 | PPC,SOM,150um | 0.028 | 0.004 |
| 17 | AC,Non-SOM,180um | 0.012 | 0.001 |
| 18 | PPC,Non-SOM,180um | 0.019 | 0.001 |
| 19 | AC,SOM,180um | 0.019 | 0.006 |
| 20 | PPC,SOM,180um | 0.027 | 0.004 |
| 21 | AC,Non-SOM,210um | 0.016 | 0.001 |
| 22 | PPC,Non-SOM,210um | 0.025 | 0.001 |
| 23 | AC,SOM,210um | 0.012 | 0.006 |
| 24 | PPC,SOM,210um | 0.025 | 0.004 |
| 25 | AC,Non-SOM,240um | 0.020 | 0.001 |
| 26 | PPC,Non-SOM,240um | 0.029 | 0.001 |
| 27 | AC,SOM,240um | 0.027 | 0.006 |
| 28 | PPC,SOM,240um | 0.036 | 0.004 |
| 29 | AC,Non-SOM,270um | 0.021 | 0.001 |
| 30 | PPC,Non-SOM,270um | 0.035 | 0.001 |
| 31 | AC,SOM,270um | 0.017 | 0.006 |
| 32 | PPC,SOM,270um | 0.034 | 0.005 |
| 33 | AC,Non-SOM,300um | 0.025 | 0.001 |
| 34 | PPC,Non-SOM,300um | 0.037 | 0.001 |
| 35 | AC,SOM,300um | 0.016 | 0.007 |
| 36 | PPC,SOM,300um | 0.037 | 0.005 |
| 37 | AC,Non-SOM,330um | 0.026 | 0.002 |
| 38 | PPC,Non-SOM,330um | 0.043 | 0.001 |
| 39 | AC,SOM,330um | 0.019 | 0.007 |
| 40 | PPC,SOM,330um | 0.044 | 0.006 |
| 41 | AC,Non-SOM,360um | 0.026 | 0.002 |
| 42 | PPC,Non-SOM,360um | 0.042 | 0.002 |
| 43 | AC,SOM,360um | 0.018 | 0.008 |
| 44 | PPC,SOM,360um | 0.038 | 0.006 |
| 45 | AC,Non-SOM,390um | 0.028 | 0.002 |
| 46 | PPC,Non-SOM,390um | 0.046 | 0.002 |
| 47 | AC,SOM,390um | 0.023 | 0.009 |
| 48 | PPC,SOM,390um | 0.039 | 0.008 |
| 49 | AC,Non-SOM,420um | 0.028 | 0.002 |
| 50 | PPC,Non-SOM,420um | 0.042 | 0.002 |
| 51 | AC,SOM,420um | 0.025 | 0.011 |
| 52 | PPC,SOM,420um | 0.040 | 0.009 |
| 53 | AC,Non-SOM,450um | 0.024 | 0.003 |
| 54 | PPC,Non-SOM,450um | 0.045 | 0.003 |
| 55 | AC,SOM,450um | 0.033 | 0.014 |
| 56 | PPC,SOM,450um | 0.044 | 0.011 |
| 57 | AC,Non-SOM,480um | 0.029 | 0.003 |
| 58 | PPC,Non-SOM,480um | 0.047 | 0.004 |
| 59 | AC,SOM,480um | 0.020 | 0.018 |
| 60 | PPC,SOM,480um | 0.042 | 0.014 |
| 61 | AC,Non-SOM,510um | 0.027 | 0.003 |
| 62 | PPC,Non-SOM,510um | 0.062 | 0.004 |
| 63 | AC,SOM,510um | 0.028 | 0.017 |
| 64 | PPC,SOM,510um | 0.030 | 0.013 |

**Posthoc Multiple Comparisons Results (Less relevant comparisons removed for brevity)**

| **Group A** | **Group B** | **Lower Bound** | **Estimated Difference** | **Upper Bound** | **p-value** |
| --- | --- | --- | --- | --- | --- |
| 1 | 2 | -0.098 | -0.087 | -0.075 | 9.59E-06 |
| 1 | 3 | -0.013 | 0.023 | 0.059 | 0.9453525921 |
| 1 | 4 | -0.107 | -0.078 | -0.050 | 9.59E-06 |
| 1 | 5 | 0.150 | 0.162 | 0.173 | 9.59E-06 |
| 1 | 6 | 0.120 | 0.130 | 0.141 | 9.59E-06 |
| 1 | 7 | 0.138 | 0.167 | 0.196 | 9.59E-06 |
| 1 | 8 | 0.091 | 0.114 | 0.138 | 9.59E-06 |
| 2 | 3 | 0.074 | 0.110 | 0.145 | 9.59E-06 |
| 2 | 4 | -0.020 | 0.008 | 0.036 | 1 |
| 2 | 5 | 0.238 | 0.248 | 0.258 | 9.59E-06 |
| 2 | 6 | 0.208 | 0.217 | 0.226 | 9.59E-06 |
| 2 | 7 | 0.225 | 0.253 | 0.282 | 9.59E-06 |
| 2 | 8 | 0.178 | 0.201 | 0.224 | 9.59E-06 |
| 2 | 9 | 0.305 | 0.315 | 0.324 | 9.59E-06 |
| 3 | 4 | -0.145 | -0.102 | -0.058 | 9.59E-06 |
| 3 | 5 | 0.103 | 0.139 | 0.174 | 9.59E-06 |
| 3 | 6 | 0.072 | 0.107 | 0.142 | 9.59E-06 |
| 3 | 7 | 0.099 | 0.144 | 0.188 | 9.59E-06 |
| 3 | 8 | 0.050 | 0.091 | 0.132 | 9.59E-06 |
| 3 | 9 | 0.170 | 0.205 | 0.240 | 9.59E-06 |
| 4 | 5 | 0.212 | 0.240 | 0.268 | 9.59E-06 |
| 4 | 6 | 0.181 | 0.209 | 0.236 | 9.59E-06 |
| 4 | 7 | 0.207 | 0.245 | 0.284 | 9.59E-06 |
| 4 | 8 | 0.158 | 0.193 | 0.228 | 9.59E-06 |
| 5 | 6 | -0.040 | -0.031 | -0.022 | 9.59E-06 |
| 5 | 7 | -0.023 | 0.005 | 0.034 | 1 |
| 5 | 8 | -0.070 | -0.047 | -0.024 | 9.59E-06 |
| 6 | 7 | 0.008 | 0.036 | 0.065 | 0.0002 |
| 6 | 8 | -0.038 | -0.016 | 0.007 | 0.8320 |
| 6 | 9 | 0.089 | 0.098 | 0.106 | 9.59E-06 |
| 7 | 8 | -0.088 | -0.052 | -0.017 | 1.15E-05 |
| 9 | 10 | -0.017 | -0.009 | -0.001 | 0.004083 |
| 9 | 11 | -0.034 | -0.008 | 0.019 | 1.000000 |
| 9 | 12 | -0.041 | -0.022 | -0.002 | 0.011075 |
| 9 | 13 | 0.008 | 0.017 | 0.026 | 0.000010 |
| 9 | 14 | 0.005 | 0.013 | 0.021 | 0.000010 |
| 9 | 15 | -0.007 | 0.018 | 0.043 | 0.784965 |
| 9 | 16 | -0.019 | 0.001 | 0.020 | 1.000000 |
| 10 | 11 | -0.025 | 0.002 | 0.028 | 1.000000 |
| 10 | 12 | -0.032 | -0.012 | 0.007 | 0.960780 |
| 10 | 13 | 0.019 | 0.026 | 0.034 | 0.000010 |
| 10 | 14 | 0.016 | 0.023 | 0.030 | 0.000010 |
| 10 | 15 | 0.003 | 0.027 | 0.052 | 0.008952 |
| 10 | 16 | -0.009 | 0.010 | 0.029 | 0.999184 |
| 11 | 12 | -0.046 | -0.014 | 0.018 | 0.999997 |
| 11 | 13 | -0.002 | 0.025 | 0.051 | 0.133744 |
| 11 | 14 | -0.005 | 0.021 | 0.047 | 0.496362 |
| 11 | 15 | -0.010 | 0.026 | 0.061 | 0.769850 |
| 11 | 16 | -0.023 | 0.008 | 0.040 | 1.000000 |
| 12 | 13 | 0.019 | 0.039 | 0.058 | 9.59E-06 |
| 12 | 14 | 0.016 | 0.035 | 0.054 | 9.59E-06 |
| 12 | 15 | 0.009 | 0.040 | 0.070 | 0.000207 |
| 12 | 16 | -0.004 | 0.022 | 0.049 | 0.318831 |
| 12 | 64 | -0.035 | 0.021 | 0.076 | 1.000000 |
| 13 | 14 | -0.011 | -0.004 | 0.004 | 0.999965 |
| 13 | 15 | -0.024 | 0.001 | 0.026 | 1.000000 |
| 13 | 16 | -0.036 | -0.016 | 0.003 | 0.324978 |
| 14 | 15 | -0.020 | 0.005 | 0.029 | 1.000000 |
| 14 | 16 | -0.032 | -0.013 | 0.006 | 0.900593 |
| 15 | 16 | -0.048 | -0.017 | 0.013 | 0.99428 |
| 15 | 17 | -0.026 | -0.001 | 0.024 | 1.00000 |
| 17 | 18 | -0.014 | -0.007 | 0.000 | 0.12237 |
| 17 | 19 | -0.032 | -0.008 | 0.017 | 1.00000 |
| 17 | 20 | -0.033 | -0.015 | 0.004 | 0.47611 |
| 18 | 19 | -0.025 | -0.001 | 0.023 | 1.00000 |
| 18 | 20 | -0.026 | -0.008 | 0.010 | 1.00000 |
| 18 | 21 | -0.005 | 0.002 | 0.010 | 1.00000 |
| 19 | 20 | -0.037 | -0.007 | 0.022 | 1.00000 |
| 21 | 22 | -0.016 | -0.009 | -0.001 | 0.00198 |
| 21 | 23 | -0.020 | 0.005 | 0.029 | 1.00000 |
| 21 | 24 | -0.027 | -0.008 | 0.010 | 0.99999 |
| 22 | 23 | -0.011 | 0.013 | 0.037 | 0.99779 |
| 22 | 24 | -0.018 | 0.000 | 0.019 | 1.00000 |
| 23 | 24 | -0.043 | -0.013 | 0.017 | 1.00000 |
| 25 | 26 | -0.016 | -0.009 | -0.001 | 0.00233 |
| 25 | 27 | -0.033 | -0.007 | 0.019 | 1.00000 |
| 25 | 28 | -0.035 | -0.016 | 0.003 | 0.31873 |
| 26 | 27 | -0.024 | 0.002 | 0.028 | 1.00000 |
| 26 | 28 | -0.026 | -0.007 | 0.011 | 1.00000 |
| 27 | 28 | -0.040 | -0.009 | 0.022 | 1.00000 |
| 29 | 30 | -0.021 | -0.014 | -0.006 | 0.00001 |
| 29 | 31 | -0.022 | 0.004 | 0.030 | 1.00000 |
| 29 | 32 | -0.032 | -0.013 | 0.007 | 0.93777 |
| 30 | 31 | -0.008 | 0.018 | 0.044 | 0.87804 |
| 30 | 32 | -0.018 | 0.001 | 0.020 | 1.00000 |
| 31 | 32 | -0.048 | -0.017 | 0.015 | 0.99907 |
| 33 | 34 | -0.021 | -0.013 | -0.004 | 0.00001 |
| 33 | 35 | -0.020 | 0.009 | 0.037 | 1.00000 |
| 33 | 36 | -0.032 | -0.012 | 0.008 | 0.99095 |
| 34 | 35 | -0.007 | 0.021 | 0.050 | 0.69408 |
| 34 | 36 | -0.020 | 0.001 | 0.021 | 1.00000 |
| 35 | 36 | -0.055 | -0.021 | 0.013 | 0.98091 |
| 37 | 38 | -0.026 | -0.017 | -0.008 | 0.00001 |
| 37 | 39 | -0.024 | 0.006 | 0.037 | 1.00000 |
| 37 | 40 | -0.042 | -0.018 | 0.005 | 0.56449 |
| 38 | 39 | -0.007 | 0.023 | 0.054 | 0.60811 |
| 38 | 40 | -0.025 | -0.002 | 0.022 | 1.00000 |
| 39 | 40 | -0.062 | -0.025 | 0.012 | 0.91682 |
| 41 | 42 | -0.026 | -0.016 | -0.006 | 0.00001 |
| 41 | 43 | -0.027 | 0.008 | 0.042 | 1.00000 |
| 41 | 44 | -0.038 | -0.012 | 0.014 | 0.99999 |
| 42 | 43 | -0.011 | 0.024 | 0.058 | 0.87381 |
| 42 | 44 | -0.022 | 0.004 | 0.030 | 1.00000 |
| 42 | 45 | 0.004 | 0.014 | 0.025 | 0.00007 |
| 42 | 46 | -0.014 | -0.004 | 0.007 | 1.00000 |
| 43 | 44 | -0.062 | -0.020 | 0.023 | 0.99998 |
| 43 | 45 | -0.044 | -0.010 | 0.025 | 1.00000 |
| 43 | 46 | -0.062 | -0.028 | 0.007 | 0.52356 |
| 44 | 45 | -0.016 | 0.010 | 0.036 | 1.00000 |
| 44 | 46 | -0.034 | -0.008 | 0.018 | 1.00000 |
| 45 | 46 | -0.029 | -0.018 | -0.007 | 0.00001 |
